# Subcritical Water Extraction Enables the Production of Cichoric and Caftaric Acid-Standardized *Echinacea purpurea* Root Extracts with Defined Composition and Favorable Biological Properties

**DOI:** 10.3390/molecules31132351

**Published:** 2026-07-03

**Authors:** Petko Denev, Desislava Teneva, Manol Ognyanov, Mariya Pimpilova, Ani Petrova, Georgi Dimitrov, Bela Vasileva, Kamelia Hristova-Panusheva, Natalia Krasteva, George Miloshev, Milena Georgieva

**Affiliations:** 1 Laboratory of Biologically Active Substances, Institute of Organic Chemistry with Centre of Phytochemistry, Bulgarian Academy of Sciences, 4000 Plovdiv, Bulgaria; desislava.teneva@orgchm.bas.bg (D.T.); manol.ognyanov@orgchm.bas.bg (M.O.); mariya.pimpilova@orgchm.bas.bg (M.P.); ani.petrova@orgchm.bas.bg (A.P.); georgi.dimitrov@orgchm.bas.bg (G.D.); 2 Institute of Molecular Biology “Acad. R. Tsanev”, Bulgarian Academy of Sciences, 1113 Sofia, Bulgaria; byvasileva@gmail.com (B.V.); karamolbiol@gmail.com (G.M.); milenageorgy@gmail.com (M.G.); 3 Institute of Biophysics and Biomedical Engineering, Bulgarian Academy of Sciences, 1113 Sofia, Bulgaria; kameliahristova@abv.bg (K.H.-P.); natalia.krasteva@yahoo.com (N.K.)

**Keywords:** subcritical water extraction, *Echinacea purpurea* root, hydroxycinnamic acids thermal decomposition, extract standardization, plant cell wall degradation

## Abstract

This study investigates subcritical water extraction (SWE) as an alternative to hydroalcoholic extraction for the production of *Echinacea purpurea* root extracts standardized to hydroxycinnamic acids (cichoric and caftaric acids). Extractions were performed at 100 °C, 125 °C, 150 °C, and 170 °C for 10–30 min. The recovery of cichoric and caftaric acids was significantly (*p* < 0.05) influenced by extraction temperature, with the highest values obtained within the range of 100–125 °C. Further experiments identified 110 °C for 10 min as the optimal condition, yielding the highest cumulative recovery of cichoric and caftaric acids (1.87 ± 0.10% of dry material). In the resulting dry extracts, SWE at 100–125 °C produced hydroxycinnamic acid contents of 5.5–7.1%, whereas the total dry extract yield in-creased from 24–28% at 100 °C to 40–41% at 150–170 °C (*p* < 0.05). Higher temperatures, however, reduced cichoric and caftaric acid cumulative content to 0.6–1.7% (*p* < 0.05), indicating a degradation of the target compounds. In contrast, total polyphenol recovery in-creased continuously with temperature, reaching 4.86% at 170 °C for 30 min. This was accompanied by marked increases in rutin, gallic and caffeic acid, reaching 458.5 mg/100 g dry weight (DW), 175.5 mg/100 g DW and 945.7 mg/100 g DW (*p* < 0.05), respectively, suggesting the release of bound phenolics following partial disruption of plant cell wall structures. SWE also enhanced the extraction of carbohydrates, uronic acids, fructans, proteins and organic acids, demonstrating an extensive temperature-dependent modification of the root matrix. 5-HMF was not detected in extracts obtained below 125 °C, but increased progressively at higher temperatures, reaching 200 mg/100 g (*p* < 0.05) at 170 °C. Biological evaluation in the human colorectal adenocarcinoma cell line (HT29) showed favorable cytocompatibility of SWE extracts, confirmed by cell viability, morphological assessment and low DNA damage in the Comet Assay. Overall, SWE enables the production of cichoric and caftaric acid-standardized *E. purpurea* extracts without organic solvents, supporting its application in pharmaceutical, nutraceutical, food and cosmeceutical products.

## 1. Introduction

Medicinal plants represent a key source of biologically active compounds widely utilized in medicine and pharmacy. Their therapeutic value is determined by a diverse range of secondary metabolites, including phenolic compounds, alkaloids and terpenoids, which exhibit antioxidant, anti-inflammatory, antimicrobial, and immunomodulatory activities. These properties underpin the growing interest in plant-derived extracts as components of pharmaceuticals, nutraceuticals, and functional foods, particularly in the context of developing safe and standardized natural products. Among medicinal plants, species of the genus *Echinacea* (Asteraceae) are of particular importance due to their long-standing use in traditional and modern phytotherapy. Traditionally, plants of the *Echinacea* genus (*Echinacea purpurea*, *Echinacea angustifolia*, and *Echinacea pallida)* have been used in the traditional medicine of Native Americans in North America for centuries to prevent and treat infections, particularly those affecting the respiratory tract [1,2,3,4]. Currently, *Echinacea* species are widely incorporated into dietary supplements, herbal medicinal products and functional foods, where they serve as a natural source of bioactive compounds with antioxidant and immunomodulatory properties [5,6,7].

*E. purpurea* (L.) Moench is the most extensively utilized and commercially exploited species from the *Echinacea* genus due to its well-characterized phytochemical profile and documented pharmacological effects [8]. Its biological activity is attributed to a complex mixture of bioactive compounds, including hydroxycinnamic acid derivatives (such as cichoric acid and caftaric acid), alkamides, polysaccharides, etc. [6,9,10,11,12]. The most well-documented biological activities of *Echinacea* extracts are their immunomodulatory and anti-inflammatory effects. Numerous studies have demonstrated their ability to stimulate immune responses, enhance phagocytic activity, modulate cytokine production, and influence innate immune mechanisms [3,6,7,12,13,14]. In addition, *E. purpurea* extracts exhibit antioxidant and antiviral activities, largely associated with their phenolic constituents [15,16]. As a result, Echinacea-based preparations are widely used to support immune function and reduce the severity and duration of respiratory infections [2,3].

In the EU, purple coneflower root is recognized as a traditional herbal medicinal product for the relief of symptoms of common cold and as a traditional herbal medicinal product used for the relief of spots and pimples due to mild acne. According to the European Pharmacopoeia, *E. purpurea* roots must contain at least 0.5% hydroxycinnamic acids, expressed as the sum of cichoric and caftaric acids, but there are no requirements established for *E. purpurea* powdered extracts [17]. According to the US Pharmacopeia, *E. purpurea* roots must contain at least 0.5% total phenols, calculated as the sum of caftaric acid, cichoric acid and chlorogenic acid and at least 0.025% alkylamides, whereas *E. purpurea* powdered extract must contain at least 4% total phenols, calculated as the sum of caftaric acid, cichoric acid and chlorogenic acid and at least 0.025% alkylamides [18].

The development of efficient extraction techniques is crucial for obtaining standardized *E. purpurea* extracts with reproducible composition and biological activity. Conventional extraction methods, such as maceration, Soxhlet extraction, and heat reflux extraction, typically employing organic solvents, can achieve relatively high yields but are associated with long extraction times, high solvent consumption, and potential degradation of thermolabile compounds [19,20,21]. Advanced techniques, including ultrasound-assisted, microwave-assisted, and enzyme-assisted extraction, have been introduced to improve efficiency and reduce processing time [22,23,24]. In line with the principles of green chemistry, SWE has emerged as a highly promising alternative for the recovery of bioactive compounds from plant materials [21,25]. SWE employs water under elevated temperature and pressure (100–374 °C), maintaining it in the liquid state while inducing substantial changes in its physicochemical properties. As temperature increases, the dielectric constant of water decreases, resulting in reduced polarity and enhanced solubility of moderately polar and even some non-polar compounds [26,27,28]. At the same time, the viscosity and surface tension of water decrease, while diffusivity and mass transfer rates increase, facilitating the penetration of the solvent into the plant matrix and improving extraction efficiency. In addition, the elevated ionic product of water under subcritical conditions promotes hydrolysis reactions, which may contribute to the release of bound phytochemicals from plant tissues. Consequently, SWE enables the efficient extraction of a broad spectrum of bioactive constituents using water as the sole extraction medium. This approach eliminates the need for toxic organic solvents, reduces extraction time and solvent consumption, and improves the environmental sustainability and safety of the extraction process.

Standardized botanical extracts are plant extracts containing a defined amount of one or more marker compounds or bioactive constituents within a specified range, ensuring batch-to-batch consistency and predictable biological activity [29,30,31]. Standardization is a key element of quality control in herbal and plant-based products, supporting identity, purity, potency, and safety [32,33].

Following the high demand for *E. purpurea* extracts in the pharmaceutical and nutraceutical industries, numerous studies have investigated the extraction of purple coneflower roots and aerial parts using different extraction techniques and solvent systems [11,34,35,36,37,38]. However, despite the recognized advantages of SWE as an environmentally friendly technology, studies specifically addressing SWE of *E. purpurea* remain scarce, with the report by Lekar et al. representing the only available study dedicated to this approach [39]. Moreover, previous investigations have primarily focused on extraction efficiency and chemical composition, while systematic studies aimed at producing extracts standardized according to pharmacopeial requirements and evaluating their biological safety have not been reported. Consequently, important knowledge gaps remain regarding the production of pharmacopeia-compliant *E. purpurea* extracts by SWE and their biological safety. Therefore, the present study addresses this gap by, for the first time, applying SWE to *E. purpurea* roots and establishing extraction conditions that enable the production of cichoric and caftaric acid-standardized extracts compliant with pharmacopeial requirements. In addition to the target marker compounds, the influence of extraction temperature on the recovery of other phytochemical constituents, including phenolic compounds, carbohydrates, proteins, and organic acids, was evaluated. Furthermore, the biological properties of the obtained extracts were assessed through cytocompatibility and genotoxicity studies in HT29 cells. To the best of our knowledge, this is the first study combining a systematic investigation of SWE processing conditions, pharmacopeia-oriented standardization, comprehensive phytochemical characterization, and biological safety assessment of *E. purpurea* root extracts. These findings provide new insights into the development of high-quality solvent-free botanical extracts and support the application of SWE-derived *E. purpurea* preparations in pharmaceutical, nutraceutical, functional food, and cosmeceutical products.

## 2. Results and Discussion

### 2.1. Proximate Composition Analysis of E. purpurea Roots

To assess the effectiveness of SWE of cichoric and caftaric acids from *E. purpurea* roots, as well as its impact on plant cell wall structure, a detailed chemical characterization of three independent batches of the raw plant material was first performed (Table 1). This baseline analysis provides essential insight into the initial composition, enabling evaluation of extraction efficiency and treatment-induced structural changes. In addition, the analysis allows selection of raw material with a high hydroxycinnamic acid content, which is important for the development of standardized extracts and for reducing variability associated with the starting plant material.

Across the three batches of *E. purpurea* roots, carbohydrates are the predominant component, with the highest levels in EP1 (52.1%) and EP3 (49.5%), and a lower content in EP2 (43.0%). Amongst carbohydrates, cellulose (13.8–17.0%) and fructans (11.9–16.7%) are major constituents, with fructans notably highest in EP3. Cellulose, together with fructans, accounts for more than 55% of the total carbohydrates of plant material, whereas soluble sugars represent a small part (between 10 and 18%). EP2 is characterized by a higher soluble sugar content compared to the other two batches (8%). Amongst them, fructose is the major sugar, followed by sucrose and glucose. The lowest fructose content was found in EP3, which negatively correlated with the higher fructan content of the sample. As can be seen, the ligno-cellulose matrix occupies a higher proportion in the first *E. purpurea* batch, while in the other it represents a smaller amount. This is not only related to the development of the plant itself, but could also have a negative impact on the efficiency of the extraction of target compounds in this *E. purpurea* batch due to its high resistance. In addition, it is known that in purple coneflower, fructan content (mainly inulin-type) follows a distinct seasonal dynamic tied to the plant’s energy storage and survival strategy. In general, fructan levels reach their peak during the winter months, while in the spring, stored fructans are hydrolyzed into simple sugars to fuel rapid development. In late autumn, fructan production increases significantly as the plant prepares for dormancy [40,41]. As shown (Table 1), uronic acids are the second most abundant carbohydrate constituents (15–18%), which build up the pectic polysaccharides. Our analysis shows that the level of uronic acids does not vary significantly between different *E. purpurea* batches. Crude protein is the second most abundant component of purple coneflower root, and its content varies between 11 and 16.5%, peaking in EP2. Crude lipids are the smallest constituents of *E. purpurea* root and remain low and relatively constant across all batches (0.4–0.6%). The ash content of the different batches (4.8–6.0%) is within the limits set by the European Pharmacopoeia (≤9.0%). The most pronounced variation is observed in phenolic compounds: EP3 shows markedly higher total polyphenols (2516.5 mg/100 g) and cichoric and caftaric acids cumulative content (2.17%). This is reflected in its superior antioxidant activity (ORAC 672.5 µmol TE/g DW and HORAC 139.3 µmol GAE/g DW), making EP3 the most bioactive batch overall, and pointing it out as the most suitable raw material to perform experiments with subcritical water extraction.

### 2.2. Efficiency of Subcritical Water for Extraction of Hydroxycinnamic Acids and Polyphenols from E. purpurea Roots

**Figure 1:**
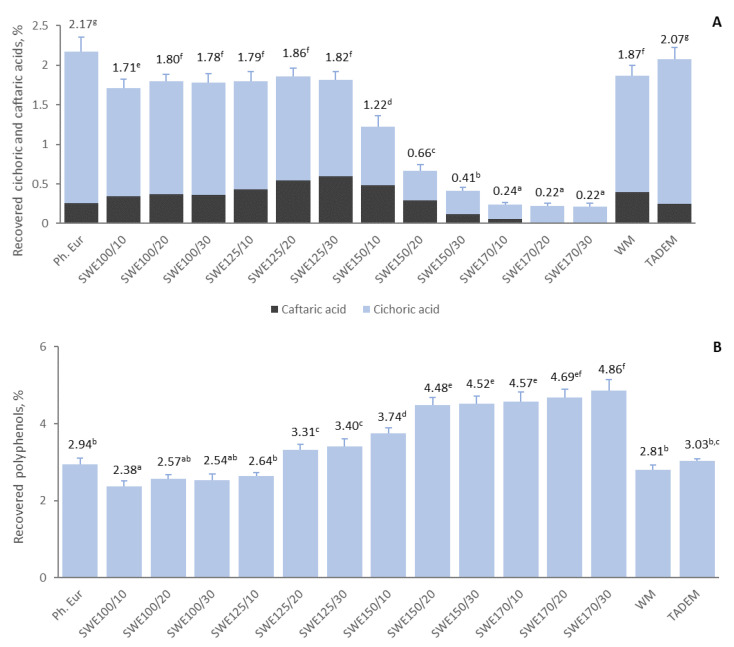
Effect of extraction method on the cumulative recovery of cichoric and caftaric acids (**A**) and polyphenols (**B**) from *E. purpurea* roots. Results are presented as mean values ± SD. There are no significant differences among values marked with the same letters within individual groups (*p* < 0.05).

In developing standardized botanical extracts, it is important to distinguish two aspects of SWE efficiency, the first being the recovery of target bioactive metabolites into the extraction medium and the second being the content of the target metabolites in the final dried extract. To assess that, we performed SWEs in twelve different combinations of temperature (100 °C, 125 °C, 150 °C and 170 °C) for three different extraction times (10 min, 20 min and 30 min) and results are presented in Figure 1. Temperature was the most influential factor governing the recovery of hydroxycinnamic acids and polyphenols from *E. purpurea* roots. Increasing the temperature up to 125 °C maintained a relatively constant cumulative amount of cichoric and caftaric acids, reaching a maximum of 1.86 ± 0.10% at 125 °C for 20 min. However, a notable compositional shift in the extracted hydroxycinnamic acids occurred at elevating temperatures. In particular, the relative content of cichoric acid in extracts decreased, while that of caftaric acid increased significantly (*p* < 0.05). For example, the ratio of the recovered individual phenolic acids was 12.0:88.0 (cichoric acid:caftaric acid) for ethanolic extractions, and 19.9:80.1, 24.2:75.8 and 39.7:60.3 for SWE100/10, SWE125/10, and SWE150/10, respectively, indicating partial cleavage of ester bonds in cichoric acid (which contains two tartaric acid moieties), leading to its conversion into simpler derivatives such as caftaric acid. At higher temperatures (up to 170 °C), a sharp decline in both total hydroxycinnamic acids and their individual components is observed. This can be attributed to thermal degradation, decarboxylation, or further breakdown of these relatively heat-sensitive compounds under subcritical water conditions. In contrast, total polyphenol content increases steadily with temperature, even at the highest levels, suggesting that a significant portion of polyphenols was initially bound to cell wall components, and elevated temperatures promote cell wall disruption and hydrolysis, enhancing their release. Additionally, the improved mass transfer and decreased solvent polarity of subcritical water at higher temperatures may further facilitate extraction. Overall, these results highlight a clear trade-off between preserving the specific hydroxycinnamic acids and maximizing total polyphenol recovery. Numerous studies have evaluated the efficiency of SWE primarily based on the recovery of broad compound groups, such as total polyphenols or total flavonoids [42]. However, the present results demonstrate that such an approach may be misleading, as these non-specific parameters do not necessarily reflect the recovery of the target bioactive constituents. In the case of *E. purpurea*, a substantial discrepancy was observed between the recovery of hydroxycinnamic acids and total polyphenols, particularly at elevated extraction temperatures. While total polyphenol content continued to increase, the concentration of cichoric and caftaric acids declined markedly due to their thermal degradation and transformation. These findings emphasize the importance of employing selective analytical approaches and monitoring specific marker compounds when developing standardized botanical extracts, rather than relying solely on global phytochemical indicators.

From the results, it is evident that the highest amounts of hydroxycinnamic acids were recovered somewhere within the interval between 100 °C and 125 °C. Therefore, to find the most favorable conditions, we performed fine-tuning of extraction parameters, performing additional extractions starting from 105 °C with 5 °C for 10 min and 20 min. The maximum recovery of cichoric and caftaric acids was achieved either at 105 °C for 20 min (1.89 ± 0.09%) or at 110 °C for 10 min (1.87 ± 0.10%) and the latter was chosen as optimal due to the reduced extraction time. At these conditions, SWE has 91% of the efficiency of ethanol extraction and was equally effective to water maceration, which was performed for a prolonged time of 45 min.

### 2.3. Characterization of E. purpurea Root Dried Extracts, Obtained by Subcritical Water in Comparison to Conventional Extraction

**Figure 2:**
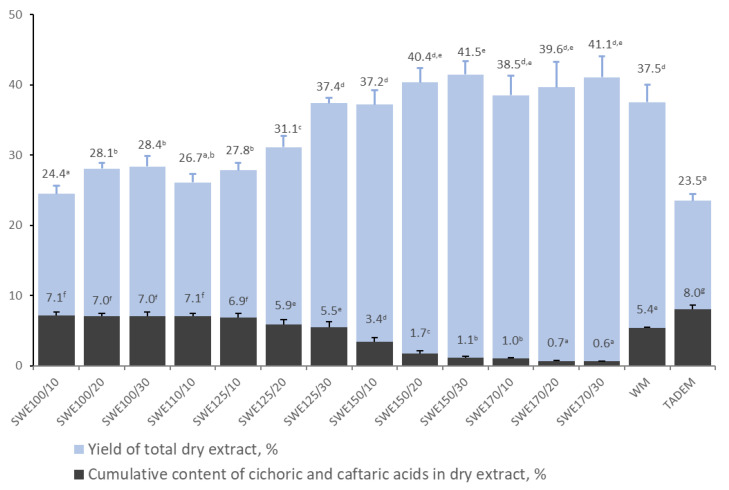
Yield of dry extract and cumulative content of cichoric and caftaric acids in dry extracts, obtained by different extraction methods. Results are presented as mean values ± SD. There are no significant differences among values marked with the same letters within individual groups (*p* < 0.05).

As already stated, the second very important aspect of SWE efficiency relates to the content of the target metabolites in the final dried extract, which is typically obtained after solvent removal and drying of the liquid extract. Therefore, it is of particular importance to analyze the content of the target metabolites, in our case caftaric and cichoric acids in the dry extracts. To this aim, SWEs were freeze-dried and analyzed. The results were compared to the temperature-assisted dynamic ethanol maceration (TADEM) and static water maceration (WM) extracts, which were freeze-dried as well (Figure 2). Both extraction yield and hydroxycinnamic acid content were strongly influenced by temperature and extraction time, with temperature again being the dominant factor. The yield of total extract increased steadily with temperature, reaching its highest values at 150–170 °C (≈40–41%), indicating enhanced solubilization and mass transfer under subcritical water conditions. In contrast, the cichoric and caftaric acid content in the dry extracts shows an opposite trend. It increases from 100 °C to a maximum at 125 °C, suggesting optimal recovery at moderate temperatures, but then declines sharply at higher temperatures (150–170 °C), dropping to as low as 0.6%.

It is known that during SWE, elevated temperatures may cause partial degradation of plant cell walls and promote the solubilization of structural polysaccharides, proteins and other macromolecules from plant tissues [43,44,45]. Our results confirmed that although higher temperatures improve overall extraction yield, they promoted degradation or transformation of hydroxycinnamic acids, reducing their concentration in the final extracts. Notably, shorter extraction times at elevated temperatures tend to preserve slightly higher levels of these acids compared to prolonged treatments, indicating time-dependent degradation effects. Compared to conventional methods (e.g., water maceration), SWE provides both higher yield and enriched hydroxycinnamic acid content, while excessive thermal input compromises extract quality. The relative content of cichoric and caftaric acids was the highest in TADEM extract—approx. 34% of the weight of the dry extract, indicating certain cell wall components (i.e., proteins, pectic polysaccharides, etc.) are not extracted with this solvent.

Although water maceration was equally efficient in recovery of hydroxycinnamic acids from the plant matrix as SWE at temperatures in the range of 100–125 °C, from Figure 2, it is evident that due to the prolonged extraction time, water maceration yields a significantly higher amount of dry extract, which results in a significantly lower amount of the sum of cichoric and caftaric acids in the dried extract (7.1% vs. 5.4%, *p* < 0.05).

In order to get more details on the changes in the chemical composition of the extracts, we performed additional analysis of the main phenolic components in the extracts, besides cichoric and caftaric acids, and their antioxidant activity (Table 2).

Caffeic acid and rutin emerged as principle phenolic components, with their concentrations varying drastically across thermal regimes, indicating that extract phenolic composition was strongly influenced by the temperature and duration of the SWE. Specifically, raising the water temperature from 100 to 150 °C leads to a more than 13-fold increase in free caffeic acid from 72.7 to 945.7 mg/100 g DW and rutin from 28.6 to 458.5 mg/100 g DW. This occurrence is likely due to the hydrolysis of ester bonds in cichoric and caftaric acids and the liberation of free caffeic acid, which supports the trend observed in Figure 1 towards decreasing the content of cichoric acid at moderate temperatures and the decrease in the total amount of hydroxycinnamic acids at higher temperatures. The amount of chlorogenic acid also decreased with the elevation of temperature indicating again the break of the ester bonds in the molecule. Another possible reason for the liberation of caffeic acid and gallic acid could be the hydrolysis of complex phenolic structures like lignin or the degradation of plant material at elevated temperatures [34,46]. Gallic acid reached its peak concentration of 175.5 mg/100 g DW at SWE170/20 [47].

Variations in phenolic profiles across temperatures reveal how compounds transform during SWE. The sharp decline in cichoric and caftaric acids at temperatures above 125 °C, accompanied by a 13-fold increase in free caffeic acid, points directly to the autohydrolysis of ester bonds mediated by subcritical water. As the temperature rises, the ionic product of water (K_w_) increases significantly, causing it to act as a pseudo-acid catalyst that cleaves the ester linkages of cichoric and caftaric acids, subsequently liberating free caffeic acid [48]. However, this phenomenon occurs in three consecutive stages: extraction, hydrolysis, and thermal degradation. This is evident at 170 °C, where caffeic acid drops abruptly to 8.8 mg/100 g DW due to thermally induced decarboxylation into pyrolytic sub-products (such as 4-vinylcatechol). A similar hydrolysis mechanism governs the behavior of rutin, which peaks at 150 °C 458.5 mg/100 g DW before undergoing cleavage of its glycosidic bond and subsequent heterocyclic ring-opening at 170 °C [49]. In contrast, gallic acid exhibits its maximum concentration at the highest thermal regime (SWE170/20, 175.5 mg/100 g DW), indicating that severe conditions are required to depolymerize matrix-bound phenolics and disrupt resilient covalent bonds within the cell wall network [50].

The increased amount of phenolic compounds possessing more free (non-esterified) hydroxyl groups resulted in elevated peroxyl radical scavenging values, indicating that antioxidant properties of the *E. purpurea* extracts could be influenced by the individual phenolic profile and the specific chemical transformations occurring at different extraction temperatures [51]. Crucially, the antioxidant activity does not decrease linearly with the degradation of native hydroxycinnamic acids, maintaining peak values at SWE150/30 and SWE170/20. This trends can be attributed to two simultaneous phenomena: first, the newly liberated free phenolic monomers possess lower steric hindrance and higher radical scavenging efficiency than their esterified precursors; second, at temperatures between 150 °C and 170 °C, co-extracted carbohydrates and proteins undergo thermal interconversions via the Maillard reaction, generating neo-formed soluble products (such as melanoidins) with potent intrinsic antioxidant properties [25].

### 2.4. Influence of Subcritical Water Extraction on Primary and Secondary Cell Wall Constituents of E. purpurea Root

To understand and evaluate how subcritical water impacts the plant material, we analyzed the protein, cellulose, uronic acid, fructan, and lignin levels in the solid extraction residues. The data in Table 3 shows that both extraction time and temperature alter the primary cell wall components. Protein levels in the extraction residues remained unchanged (12–13%). However, based on the yield of residue (residue yield), only 38% of the original crude protein remained in the SWE150/20 residue, suggesting that a significant portion of the initial proteins (>60%) is already present in the extract. In contrast, the milder SWE treatment (100/10 and 100/20) left 55% of the proteins in the residue, meaning that 45% passed into the extract. Evidence indicates that proteins are generally more stable than some carbohydrate polymers, only showing significant breakdown at temperatures above 190 °C. Researchers suggest that heat-induced denaturation and clumping (between 40 and 80 °C) actually improve protein stability by making them less soluble [52]. While higher temperatures eventually break proteins down into peptides and amino acids, studies show that 150 °C for 15 min is sufficient to extract maximum protein from sunflower by-products without causing degradation [53].

Uronic acids, which comprise acidic polysaccharides like pectin, followed a similar pattern. The levels of uronic acids in the residues dropped by 33% and 50% after SWE treatment at 150/10 and 150/20, respectively, showing that a significant portion was extracted. Notably, only 34% of the initial uronic acids remained in the SWE150/20 residue, meaning 66% were solubilized and removed. Under milder extraction conditions (100/10 or 100/20), however, the cell walls remained largely intact, as the residues retained most of their initial uronic acid content. By contrast, the results in Table 3 suggest that fructans were much more easily extracted and affected by SWE conditions than other cell wall components. Under harsher conditions, fructan content in the residues was reduced more than 2–3 times (2–3%). Interestingly enough, not more than 37% of the initial fructans were recovered in the residue after a 100/10 SWE, showing that 63% can be found in the extract. It is interesting to note that the 150/20 residue contained not more than 10% of the initial total fructan content, expecting >90% to be found in the extract. It should be mentioned that a greater extent of decomposition of fructans to different intermediates was achieved at a significantly lower temperature (100 °C) [54]. Cellulose, the structural backbone of plant cell walls and the primary component of lignocellulose, remained largely unaffected by SWE, showing much higher stability than uronic acids, proteins, and fructans. In residues, 98–108% of the initial cellulose was retained, indicating minimal impact and poor solubilization at the employed SWE conditions. Significant breakdown of the cellulose structure only began under more intense conditions (>350 °C) [55]. Similar to cellulose, SWE treatment led to an increase in acid-insoluble lignin within the residues, peaking at nearly 21% in the SWE150/20 residue. Unlike some polysaccharide constituents of *E. purpurea* root, which suffered some degradation, acid-insoluble lignin remained virtually unchanged by the milder treatment, similar to cellulose. This stability is confirmed by the recovery rates, with SWE100/10 residue retaining 101% of the initial lignin found in the purple coneflower root. Under harsher extraction conditions (150/10 and 150/20), however, it seemed that lignin degradation was initiated, since the residues retained a smaller amount of their initial lignin content (88–90%). It is quite possible that under SWE acidic conditions, a minor portion of acid-soluble lignin was dissolved, while the majority of lignin typically forms an insoluble precipitate and was retained in the residue. Regarding the degradation of lignin under SWE conditions, a previous study noted that it began to break down in naked oat stems at 170 °C. The process peaked at 190 °C, where nearly 50% of the lignin was removed through autohydrolysis [56].

Table 4 presents data for the uronic acids content of the extracts, as well as the total carbohydrate and organic acid contents. The lyophilized extracts were characterized by total carbohydrate content in the range of 40–57%. The lack of a clear trend in total carbohydrate content may reflect the highly complex and variable composition of the extracts across different preparation conditions. This is further complicated by the limited specificity, the difficulty of standardizing the analytical method used to measure carbohydrate concentrations (glucose equivalents), and varying molar absorption coefficients that depend on the chemical reactivity of carbohydrates with the derivatization reagent.

Concerning uronic acids, an increase in both the temperature and the duration of the extraction process resulted in an increase in their content in the extracts from 2.8 to 6.2% (SWE125/20). This suggests that pressurized hot water enhances the diffusion rate and solubility of uronic acid-containing polysaccharides (e.g., pectin). However, raising the temperature further and especially the extraction time led to a gradual decrease in content (2.5% in SWE170/30). An interesting comment could be made by further analysis of recovery data and information in Table 3. Based on the uronic acid content (Table 3 and Table 4), the SWE100/10 and 100/20 extracts contained only a minor part of the initial uronic acids—about 8% and 14% (Table 4). Theoretically, this implies that nearly 92% and 86% of the initial uronic acids should remain in the residues after extraction. Results obtained confirmed this statement. The data from Table 3 show that residues from SWE100/10 and SWE100/20 experiments retained 91% and 85% of the initial uronic acids, respectively. This is not the case when considering extracts obtained at higher temperatures and for longer durations. For example, the SWE150/10 and SWE150/20 extracts had uronic acid contents of 4.6% and 4.3%, respectively, representing 20% of the raw material’s initial uronic acid content. These findings indicate that the residues should retain 80% of the initial uronic acid content. However, data from Table 3 show that residues from SWE150/10 and SWE150/20 recovered only 49% and 34% of the initial uronic acids, respectively. This discrepancy indicates that 31% and 46% of the uronic acid constituents underwent degradation (decarboxylation, dehydration, etc.) into forms that could not be estimated.

In Table 4, the free sugar composition pattern is also included. It can be seen that fructose and glucose levels were stable at lower temperatures (<125 °C and times of 10, 20 min), but higher temperatures caused a gradual increase in their concentration. Sucrose concentration remained stable under these conditions (<150 °C/10 min). Fructose and glucose concentration in dried extracts peaked at SWE150/20 and negatively correlated with the sucrose level. However, prolonged extraction times (SWE150/30) further reduced both disaccharide and monosaccharides glucose and fructose amounts, likely due to thermal degradation. Notably, fructose levels significantly exceeded those of sucrose, indicating that inulin-type fructans, rather than just sucrose, were the primary source of fructose during breakdown. The results obtained revealed that fructose decomposed much faster than glucose. At 150 °C for 30 min, sucrose levels dropped by nearly 20 times. Beyond these conditions, higher temperatures and longer extraction times resulted in the near-total disappearance of sucrose from the extracts. Different studies showed that a pronounced conversion of sucrose into fructose and glucose occurs more rapidly at acidic conditions for short residence times [57,58]. It is known that the Maillard reaction between reducing sugars and amino compounds generates intermediates that can further degrade into compounds such as 5-HMF and furfural, ultimately leading to the formation of melanoidins. In addition, monosaccharides, particularly fructose, are highly susceptible to acid-catalyzed and thermal degradation under elevated temperatures, resulting in the formation of 5-HMF, furfural, organic acids, and other intermediate decomposition products [59]. There were clear indications for such a process, examining organic acids results (Table 4). For example, higher temperatures caused a gradual increase in acetic acid levels, having a maximum value at 150 °C. The amount of oxalic acid, a fragmentation product of furfural derivatives, was observed to decline with increasing temperature and time of extraction. It is important to note that oxalic acid is thermally unstable at high temperatures (125–175 °C), which probably explains the decrease in its amount with the increase of temperature. Other organic acids, such as malic and citric acids, can also be formed as a result of amino acid decomposition (decarboxylation, deamination, etc.). This could explain the considerable increase in their levels at temperatures exceeding 150 °C and treatment longer than 10 min. A review of the scientific literature on the topic shows that aspartic acid primarily degrades via deamination into maleic and fumaric acids. Subsequent rehydration forms malic acid, a precursor to pyruvic acid, which ultimately yields acetic or lactic acid [60]. However, the quantification of tartaric acid, a key degradation product of caftaric and cichoric acids, was not feasible due to interference from a large matrix-derived peak that overlapped with its chromatographic signal, representing a limitation of the present study. In order to gain a better insight into the breakdown products and changes in carbohydrate components during extraction, we analyzed the resulting extracts for 5-hydroxymethylfurfural content. The results are also included in Table 4. The presence of 5-HMF revealed that carbohydrate constituents (mainly fructose, sucrose, and inulin) were actively decomposed at higher temperatures. The table data indicate that 5-HMF was undetectable in extracts obtained below 125 °C. However, higher extraction temperatures led to a progressive increase in its content, reaching a peak of 201 mg/100 g at 170 °C. It seemed that the decomposition of carbohydrate constituents began at temperatures higher than 125 °C. Our findings are in line with those of earlier studies, which demonstrated that significant accumulation of 5-HMF begins between 120 and 140 °C [61]. Below 140 °C, formation is negligible. However, yields increase sharply, peaking between 180 and 200 °C [62,63]. The 5-HMF levels obtained in this study were comparable to those reported in the previous literature. Fan and Gao observed that 5-HMF content increased from 200 mg/100 g at 160 °C to a peak of 430 mg/100 g at 200 °C [61]. In contrast, our results were lower than the values reported by Herrero et al. (~300 mg/100 g at 175 °C) [62], but in close agreement with the findings of Natolino et al. [64], who reported 5-HMF concentrations ranging from 31 to 239 mg/100 g in extracts obtained between 120 and 200 °C.

### 2.5. Comparative Biological Evaluation of E. purpurea Root Extracts Obtained by Subcritical Water and Conventional Extraction Methods

The biological activity of four *E. purpurea* extracts obtained by SWE (SWE100/10, SWE125/10, SWE150/10, and SWE170/10) was comparatively evaluated against conventionally extracted fractions (WM and TADEM) in HT29 colorectal adenocarcinoma cells following 24–48 h exposure at concentrations of 100–200 µg/mL. A multiparametric analysis was performed, including cell viability, cellular morphology and DNA damage assessment (Comet Assay), enabling a comprehensive comparison of extract performance.

#### 2.5.1. Effects of *E. purpurea* Extracts on Cell Viability

**Figure 3:**
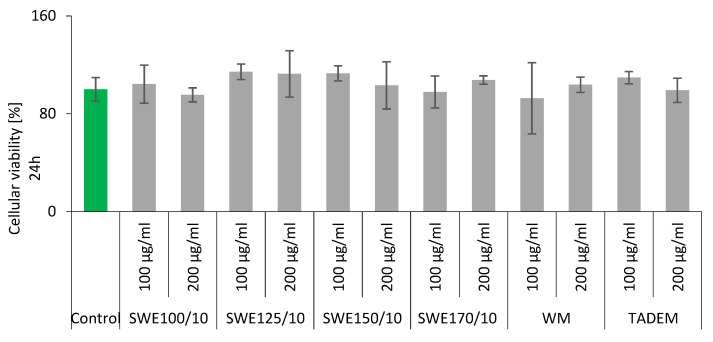
Cell viability of HT29 cells following 24 h and 48 h treatment with SWE100/10, SWE125/10, SWE150/10, SWE170/10, WM and TADEM extracts at 100 and 200 μg/mL, determined by WST-1 assay. Results are expressed as percentage of viability relative to untreated control cells (set as 100%) and presented as mean ± SD of three independent experiments performed in replicates.

Figure 3 demonstrates the comparative cytocompatibility and time-dependent antiproliferative effects of subcritical water versus conventionally extracted fractions. One of the most notable observations in this study is the higher cytocompatibility of the SWE fractions, particularly during the early treatment period. At 24 h, several SWEs maintained or slightly increased HT29 metabolic activity relative to untreated controls, whereas the conventionally obtained WM fraction reduced viability, indicating a less favorable short-term cytocompatibility profile (Figure 3). Among all tested samples, SWE125/10 showed the strongest positive effect on cellular metabolic activity, reaching 114.34% viability at 100 µg/mL and 112.66% at 200 µg/mL. Similarly, SWE150/10 maintained high viability values of 113.03% and 103.26%, respectively, while SWE100/10 preserved cell viability close to control levels (104.25% at 100 µg/mL). SWE170/10 also demonstrated a moderate but stable effect. In contrast, the conventional extract WM reduced viability to 92.67% at 100 µg/mL, whereas TADEM remained near baseline but did not outperform the SWE fractions. These findings suggest that the extraction strategy plays an important role in preserving functionally active phytochemicals. The results are consistent with previous reports indicating that aqueous and subcritical extraction approaches can improve the recovery of bioactive phenolics and phytoconstituents while reducing solvent-associated degradation [65,66].

#### 2.5.2. Morphological Assessment of HT29 Cells Following Extract Treatment

**Figure 4:**
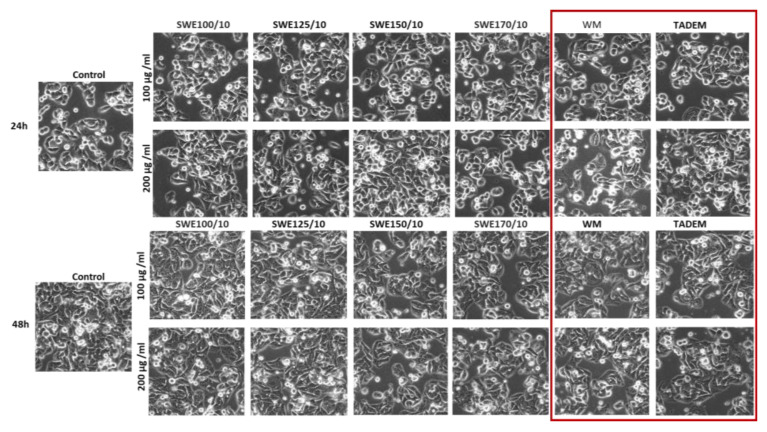
Morphological changes in HT29 cells following treatment with *E. purpurea* after 24 h and 48 h exposure to SWE100/10, SWE125/10, SWE150/10, SWE170/10, WM and TADEM at final concentrations of 100 and 200 μg/mL. The scale bar is 100 µm.

Morphological changes in HT29 cells were evaluated after 24 h and 48 h of treatment with six *E. purpurea* extracts. Extracts were applied at final concentrations of 100 µg/mL and 200 µg/mL. Representative images are shown in Figure 4. At 24 h, untreated control cells displayed typical epithelial morphology, characterized by high confluency and tight cell–cell contacts. Cells treated with *E. purpurea* extracts exhibited largely preserved morphology, particularly at 100 µg/mL. Mild alterations were observed at 200 µg/mL, including slight cell rounding and modest reductions in confluency only in SWE170/10 and WM-treated cells. At 48 h, the morphological changes across all treatment groups were minimal. Control cells maintained a dense monolayer, whereas treated groups, mostly those treated with conventional extracts, showed slowly reduced confluency and increased intercellular spaces, likely due to partial cell detachment.

The morphological assessment further supported the favorable biocompatibility profile of SWE-derived extracts. The limited structural alterations observed in SWE-treated cells indicate that these fractions do not induce pronounced membrane damage or loss of adhesion. In contrast, conventional extracts produced more noticeable reductions in confluency and increased signs of cellular detachment, suggesting a greater stress burden on treated cells. Taken together, these observations indicate that SWE extracts preserve cellular structural integrity more effectively than conventional fractions.

#### 2.5.3. DNA Damage and Genomic Stability (Comet Assay)

**Figure 5:**
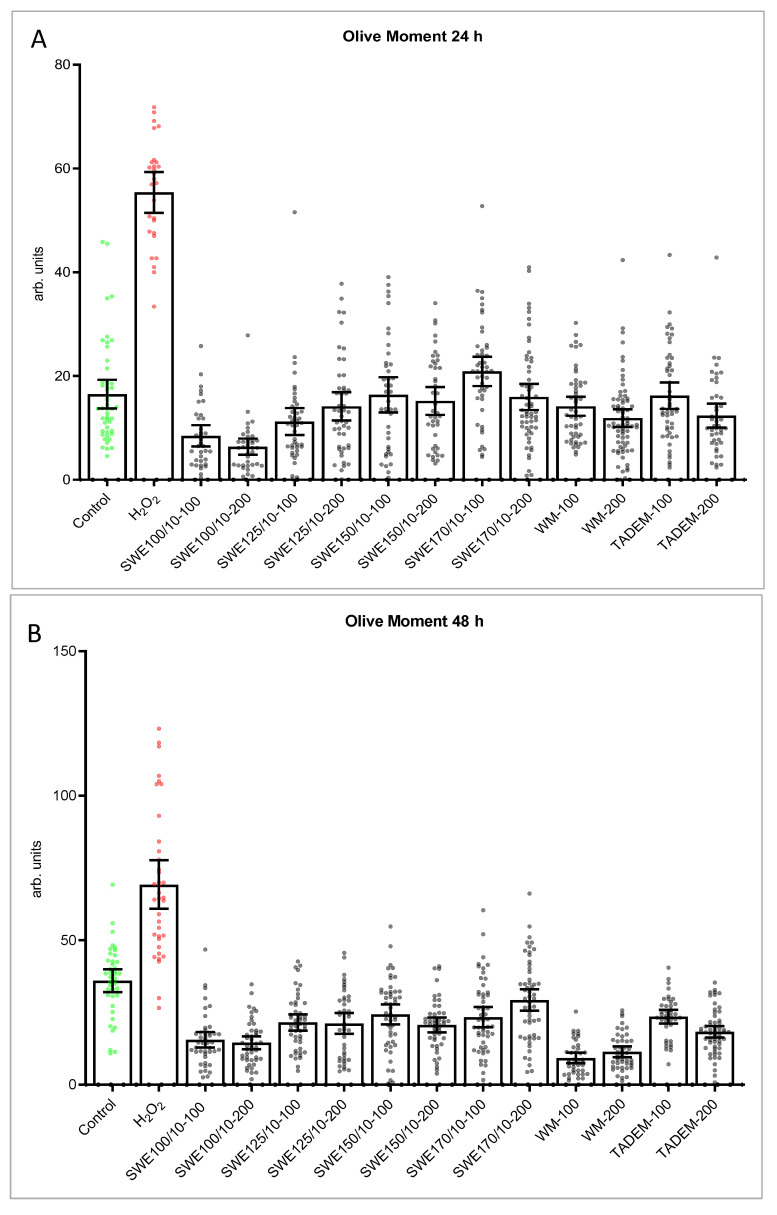
Genotoxic effects of *E. purpurea* extracts in HT29 cells assessed by Comet Assay after (**A**) 24 h and (**B**) 48 h treatment with SWE100/10, SWE125/10, SWE150/10, SWE170/10, WM, and TADEM extracts (100 and 200 μg/mL). Data are presented as mean ± SD from biological replicates. The Comet Assay quantitative analysis indicates the relative genotoxic burden induced by each extract and highlights the genomic stability profile of SWE fractions.

Genotoxicity testing using the Comet Assay further characterized the effects of *E. purpurea* extracts in HT29 cells (Figure 5). At 24 h, all SWEs produced substantially lower DNA damage than the H_2_O_2_ positive control (55.4) (Figure 5A). The most favorable response was observed for SWE100/10, with mean Olive Moment values of 8.47 (100 µg/mL) and 6.38 (200 µg/mL). SWE125/10 also demonstrated low DNA damage values (11.24–14.14), comparable to or lower than those of the conventional fractions. By comparison, WM and TADEM exhibited values ranging from 11.89 to 16.22, indicating a slightly higher genotoxic burden than the best-performing SWE fractions. At 48 h, DNA damage remained moderate in the SWE groups, with no evidence of excessive genomic instability (Figure 5B). Importantly, none of the tested extracts induced DNA damage approaching that of the positive control, indicating an overall low genotoxic risk under the applied experimental conditions.

The Comet Assay results additionally reinforce the biological advantages of SWE-derived extracts. Although all treatment groups produced substantially lower DNA damage than the positive control, several SWE fractions, particularly SWE100/10 and SWE125/10, showed lower Olive Tail Moment values compared with conventionally extracted fractions. This finding suggests that SWE-derived extracts exert their biological effects without inducing substantial genomic instability. It also supports the possibility that SWE fractions contain bioactive compounds with protective redox-modulating and DNA-stabilizing properties, as evidenced similarly in phytochemical modulation studies [65,66].

Overall, the biological evaluation demonstrated that subcritical water-derived *E. purpurea* extracts exhibit a favorable safety and cytocompatibility profile. Compared with conventionally obtained fractions, SWEs generally maintained higher metabolic activity, preserved cellular morphology, and induced only limited DNA damage, indicating that enhanced biological activity was not accompanied by increased cellular stress or genomic instability. Among the tested samples, SWE125/10 consistently showed the most favorable performance across the evaluated biological endpoints. These findings suggest that SWE not only enables the production of cichoric and caftaric acid-standardized extracts, but also contributes to preserving biologically relevant constituents associated with cellular integrity and genomic stability. Furthermore, standardization based on pharmacopeial marker compounds represents an important strategy for improving batch-to-batch consistency and quality control of botanical extracts. By establishing extraction conditions that reliably yield extracts meeting predefined hydroxycinnamic acid specifications, variability in the composition of the final product can be reduced despite the inherent variability of plant raw materials and extraction processes. Therefore, the standardization-oriented approach applied in the present study may contribute to improved reproducibility and facilitate the industrial production of SWE-derived *E. purpurea* extracts. Taken together, the phytochemical and biological findings support the potential of SWE as a green and efficient technology for obtaining high-quality, pharmacopeia-compliant *E. purpurea* extracts suitable for pharmaceutical, nutraceutical, functional food, and cosmeceutical applications. However, it should be noted that the present study has several limitations. First, although alkamides represent important pharmacopoeial and bioactive markers for *Echinacea purpurea* roots, their analysis was outside the scope of the current study, which focused primarily on the standardization of hydroxycinnamic acids in line with the European Pharmacopoeia requirements. Furthermore, the biological evaluation was performed using only a single cell line. Future studies should therefore incorporate comprehensive alkamide characterization together with additional cell line models and in vivo validation to provide a more complete assessment of the biological activity and pharmaceutical and nutraceutical potential of subcritical water-derived *E. purpurea* root extracts.

## 3. Materials and Methods

### 3.1. Plant and Analytical Materials

Three distinct batches of *E*. *purpurea* (L.) Moench roots were investigated in this study. EP1 was sourced directly from a licensed producer—Fatma Kichukova, in Gotse Delchev, Bulgaria (net weight 5 kg). EP2 was purchased from Bulgarian Bio Product Ltd. (Simitli, Bulgaria; Batch L21032025, net weight 5 kg), and EP3 was obtained from Bioprogramme EAD (Dobroslavtsi, Bulgaria; Batch L20032028, net weight 5 kg). All batches of *E. purpurea* were acquired in March 2025 and kept in paper bags at room temperature. All analytical materials, reagents, and standards for flavonoids, phenolic acids, sugars, and organic acids were purchased from Sigma-Aldrich (Darmstadt, Germany).

### 3.2. Preparation of E. purpurea Root Extracts

#### 3.2.1. Reference Extraction Procedure of Caftaric Acid and Cichoric Acid, and Other Phenolic Components

Reference extraction procedure of caftaric acid and cichoric acid was conducted in accordance with the methodology prescribed by the European Pharmacopoeia [17]. Approximately 0.5 g of finely powdered *E. purpurea* root was combined with 80 mL of a 70% (*v*/*v*) ethanol solution in a 100 mL volumetric flask. The mixture was subjected to ultrasonic extraction for 15 min, after which the volume was adjusted to 100 mL using the same solvent. Following filtration through a 0.45 µm PTFE membrane, the filtrate was analyzed for caftaric acid, cichoric acid, and other phenolic constituents.

#### 3.2.2. Static Water Maceration (WM)

For this procedure, 20 g of powdered *E. purpurea* root was mixed with 400 mL of distilled water (solid-to-liquid ratio 1:20) in round bottom glass flasks. The extraction was carried out at 90–100 °C for 45 min. After the extraction process, the mixture was filtered. The procedure was performed in duplicate, and the resulting extract was denoted as a WM extract. The sample obtained at 100 °C for 45 min was used for the subsequent determination of phenolic compounds and other bioactive constituents.

#### 3.2.3. Temperature-Assisted Dynamic Ethanol Maceration (TADEM)

For this procedure, 20 g of powdered plant material was mixed with 400 mL of 70% (*v*/*v*) ethanol (solid-to-liquid ratio 1:20) in 1 L glass bottles. The bottles were shaken in a water bath manufactured by NUVE (Asagi Ovecler, Ankara, Turkey) at 60 °C for 1 h. The bottles were then cooled, and the extract was filtered. The extraction procedure was repeated twice, and the resulting extract was denoted as the TADEM extract.

#### 3.2.4. Subcritical Water Extraction (SWE)

SWE was executed using a 2 L apparatus (InnoSolv Ltd., Plovdiv, Bulgaria) featuring an insulated extraction chamber, an electrical heating unit, and a circulation pump. To maintain the solvent in a liquid state at the selected temperatures (100 °C, 125 °C, 150 °C and 170 °C), a constant nitrogen-induced pressure of 10 bar was regulated within the system. The procedure involved loading 100 g of plant material into a metal sieve insert (1:20 solid-to-solvent ratio). Once the target temperature was stabilized, the 2 L of water was continuously circulated through the material for durations of 10, 20, or 30 min. Post-extraction, the resulting fluids were cooled in situ to ambient temperature.

Samples were labeled according to their specific extraction conditions described in Table 5.

### 3.3. Preparation of Freeze-Dried Extracts

Following SWE, WM and TADEM, the crude extracts were clarified via filtration and centrifugation (6000 rpm, 10 min). The organic solvent was eliminated through rotary evaporation at 45 °C, after which all extracts were freeze-dried using a lyophilizer (Alpha 1–4 LDplus, Martin Christ Freeze Drying Systems GmbH, Osterode am Harz, Germany). The extraction yield was calculated as the weight (g) of dry extracts obtained from 100 g of raw materials and expressed as a percentage. To prevent phytochemical degradation, the dry residues were maintained in a moisture-free, dark environment. Analytical solutions were prepared by dissolving the lyophilized material in ultra-pure water (5 mg/mL).

### 3.4. Proximate Composition Analysis of Plant Material

Moisture content was determined gravimetrically by drying the milled sample (~1.0 g) in an automated moisture analyzer (KERN DLB 160-3A, Bensheim, Germany) at 105 °C until constant weight. Ash content was determined gravimetrically by placing the pulverized sample (1.0 g) in a crucible and igniting it in a Dentamatic 6000-M muffle furnace (Tokmet-TK Ltd., Varna, Bulgaria) at 560 °C to a constant mass. Crude lipid content was estimated gravimetrically using a Randall extractor SER 148 (VELP Scientifica Srl, Usmate, Italy). Initially, the ground samples (2.0 g) were packed in a cellulose thimble (33 × 88 mm) and subjected to a petroleum ether (70 mL) two-step extraction, including immersion for 30 min, and washing for 2 h, followed by a 2 h solvent recovery phase. The crude extract was additionally dried under vacuum, and its weight was used to calculate lipid content. The temperature of the heating plate and seal type were chosen according to the manufacturer’s instructions. Crude protein content was tested by the micro-Kjeldahl method (N × 6.25). Ammonia nitrogen, in the digested sample, was determined by the acetylacetone–formaldehyde colorimetric method, using ammonium sulfate as a standard [67]. The total carbohydrate content of the roots was analyzed by the phenol-sulfuric acid method, using glucose to construct the calibration curve [68]. After solubilizing the sample in 72% (*w*/*w*) H_2_SO_4_ (1 h, 30 °C), a 3 h acid hydrolysis (100 °C, 1 M H_2_SO_4_) was further performed. The obtained hydrolysate was used as the sample for analysis. Absorbance was measured at 490 nm.

### 3.5. Uronic Acid, Cellulose, Starch, and Total Fructan Content

For the estimation of the uronic acid content of the raw material, the method of Ahmed and Labavitch was used [69]. In brief, the sample was threefold-extracted with ethanol (70% (*v*/*v*), 50 °C, 1 h). The mixture was centrifuged (18,187× *g*) to separate solid material before each repetition. In addition, the residue was soaked (2×, 1 h) with pure acetone at room temperature and vacuum-dried. Finally, the sample was hydrolyzed as described in 3.4, and the filtrated hydrolyzate was run as a sample for analysis. The hydrolysates were assayed employing the colorimetric 3-phenylphenol method according to Blumenkrantz and Asboe-Hansen [70]. The absorbance was measured at 520 nm.

Cellulose content was quantified using a modified version of Updegraff’s spectrophotometric semi-micro method [71]. Briefly, a 30–35 mg sample was gently boiled for 30 min in a microtube with an O-ring screw cap containing 2 mL of an acetic acid–nitric acid reagent (acetic acid:H_2_O:HNO_3_ 8:2:1 *v*/*v*/*v*). After colling, the solid residue was centrifuged and washed with water until it reached a neutral pH. The remaining solids were dissolved in 72% *w*/*w* sulfuric acid and, after an appropriate dilution, run via the phenol–sulfuric acid method.

The total fructan content of purple coneflower roots was estimated using a resorcinol-thiourea method of Roe [72]. Initially, the powdered root was subjected to double extraction (1:10 *w*/*v*, 20 min, 65 °C) with water in an ultrasonic bath Elmasonic p 70 H (Elma Schmidbauer GmbH, Singen, Germany) (37–80 kHz, 220 W) [73]. The combined extracts were further analyzed by the method. The resorcinol solution was prepared by dissolving 0.1 g resorcinol and 0.25 g thiourea in 100 mL of glacial acetic acid. A 30% hydrochloric acid solution was prepared separately. The working solution was then obtained by mixing hydrochloric acid and resorcinol solutions in a 7:1 (*v*/*v*) ratio. A volume of 0.5 mL of the extracts was mixed with 2 mL of the working solution. Then the mixture was incubated in a water bath at 80 °C for 10 min. After cooling, the absorbance of the samples was recorded at 515 nm against a reagent blank prepared in a similar way using distilled water. Fructose (10–50 µg/mL) was used for the construction of a calibration curve [72].

Total starch analysis was performed by a combination of the α-amylase/amyloglucosidase method for conversion of starch into glucose and the glucose oxidase/peroxidase/4-aminoantipyrine (GOPOD) method of measuring glucose quantity. The analysis was conducted according to the analytical protocol established by Hall [74].

### 3.6. Lignin Content

The lignin content in the initial *E. purpurea* batches was evaluated by the spectroscopic acetyl bromide lignin (ABL) method [75]. The Klason lignin gravimetric method (KL) was employed for the determination of lignin content in the residue after SWE [76]. Before performing the analysis, the plant material (3–4 g) was packed in nylon filter bags and successively extracted in a Randall extractor SER 148 (VELP Scientifica Srl, Usmate, Italy) using 70 mL of each solvent: water, ethanol (96% *v*/*v*), chloroform:methanol (2:1 *v*/*v*), and acetone [75]. Each solvent cycle (20 min immersion, 30 min washing) was repeated 3 times or until the resulting extract was colorless. The temperature of the heating plate and seal type were chosen according to the manufacturer’s instructions.

### 3.7. High-Performance Liquid Chromatography Analysis of Caftaric Acid and Cichoric Acid

Caftaric and cichoric acids were quantified following the procedure outlined in the European Pharmacopoeia [17]. The separation was carried out on a ProntoSIL 120-5-C18 SH (4.6 × 250 mm, 5 µm; BISCHOFF Chromatography, Leonberg, Germany) joined to a Nexera-*i* LC-2040C Plus UHPLC system (Shimadzu Corporation, Kyoto, Japan) with a UV detector set at 330 nm. The elution of the sample (10 µL) was conducted at 35 °C and a flow rate of 1.0 mL/min with the mobile phase consisting of (A)—phosphoric acid:water (1:999 *v*/*v*) and (B)—acetonitrile using the following gradient: 0 min—90% (A); 0–13 min 90% → 78% (A); 13–14 min 78% → 60% (A); 14–20 min 60% (A). The peaks identification was based on relative retention regarding the chlorogenic acid standard: caftaric acid = about 0.8; cichoric acid = about 2.3. The percentage content of each phenolic acid in the sample was calculated using the equations described in the corresponding monograph [17].

### 3.8. High-Performance Liquid Chromatography Analysis of Phenolic Components

Phenolic compounds were analyzed via UHPLC on a Shimadzu Nexera-i LC2040C Plus system (Shimadzu Corporation, Kyoto, Japan). The setup included a UV-VIS detector and a Shimadzu Shim-pack GIST C18 column (4.6 × 250 mm, 5 µm) and Shimadzu Shim-pack Guard Column Holder (10 mm) operated at 26 °C. A gradient elution was applied at 0.8 mL/min using 0.5% acetic acid (A) and acetonitrile (B), starting at 14% B, then increasing to 25% (6–30 min) and 50% (40 min). Detection was set at 280 nm with a 5 µL sample injection. Identification was validated by matching retention times against a broad range of phenolic standards (gallic acid, neochlorogenic acid, 3,4-dihydroxybenzoic acid, chlorogenic acid, catechin, vanillic acid, caffeic acid, epicatechin, *p*-coumaric acid, ferulic acid, rutin, ellagic acid, quercetin-3-*β*-glucoside, naringin, rosmarinic acid, myricetin, cinnamic acid, quercetin, luteolin, naringenin, apigenin, and kaempferol). Results were calculated based on standard calibration curves and are expressed in mg/100 g DW ± SD [77].

### 3.9. High-Performance Liquid Chromatography Analysis of Organic Acids

The quantitation of organic acids in the lyophilized extracts was conducted on a Nexera-*i* LC2040C Plus UHPLC system (Shimadzu Corporation, Kyoto, Japan) with a UV detector. The system was controlled by LabSolutions (ver. 5.98) software (Shimadzu Corp.). The separation was performed on a Shimadzu Shim-pack GIST C18 (4.6 mm × 250 mm, 5 µm) column at 25 °C and a flow rate of 1.0 mL/min. A 20 μL sample was auto-injected and eluted isocratically using a mobile phase composed of 25 mM K_2_HPO_4_ (pH adjusted to 2.4 with H_3_PO_4_). After UV detection of organic acids at 210 nm, their concentrations were calculated using a five-point calibration curve for each acid, with peak identities confirmed by comparing retention times against known standards (quinic, shikimic, fumaric, malic, acetic, citric and oxalic acids).

### 3.10. High-Performance Liquid Chromatography Analysis of Free Sugars

One gram of powdered plant material was extracted with 30 mL of water for one hour at 30 °C while being continuously shaken in a water bath. The resulting extract was used for analysis after being recovered through centrifugation at 6000× *g* for 20 min and filtration through a 0.45 μm PTFE filter. A UHPLC system Nexera-*i* LC2040C Plus (Shimadzu Corporation, Kyoto, Japan) with a binary pump and a 20 A refractive index detector was employed for analysis. Free sugar content was separated on a ZORBAX Carbohydrate column (4.6 × 150 mm, 5 μm) and a ZORBAX Reliance Cartridge guard column (Agilent Technologies, Santa Clara, CA, USA). A mobile phase composed of a mixture of acetonitrile and water (80:20 *v*/*v*) was used to elute the sample at a flow rate of 1.0 mL/min at 25 °C. A calibration curve built by plotting the peak area against the concentrations of each standard (glucose, fructose, sucrose, galactose, maltose) was used to find the concentration of sugars in the sample as detected by their retention time.

### 3.11. Total Polyphenol Content

Total polyphenols were quantified using the Folin–Ciocalteu’s reagent according to the method of Singleton and Rossi [78] using double-beam UV-Vis spectrophotometer V-730 (JASCO Corporation, Tokyo, Japan). Gallic acid was used as a calibration standard, and the results are expressed in mg gallic acid equivalents (GAE) per 100 g.

### 3.12. Antioxidant Activity Assays

Oxygen radical absorbance capacity (ORAC) activity was assessed using a FLUOstar OPTIMA (BMG Labtech, Ortenberg, Germany) microplate reader (excitation λ = 485 nm, emission λ = 520 nm), following the procedure established by Ou et al. [79] as modified by Denev et al. [80]. Trolox was used to build the calibration curve, and results were expressed in micromole Trolox equivalents (μmol TE) per gram DW ± SD (*n* = 8).

Hydroxyl radical averting capacity (HORAC) activity was determined on a FLUOstar OPTIMA (BMG Labtech, Ortenberg, Germany) microplate reader (excitation λ = 485 nm, emission λ = 520 nm), following the procedure described by Ou et al. [81]. Gallic acid was used as a standard, and results are expressed in micromole gallic acid equivalents (μmol GAE) per gram DW ± SD (*n* = 8).

### 3.13. Determination of 5-HMF Content

The 5-HMF content in the extracts was estimated using the method of Winkler with the *p*-toluidine-barbituric acid reagent as described by Zembrzuska et al. [82]. The analysis was performed using the double-beam UV-Vis spectrophotometer V-730 (JASCO Corporation, Tokyo, Japan).

### 3.14. Cell Culture and Experimental Setup

The human colorectal adenocarcinoma cell line HT29, obtained from ATCC, was used as the experimental model. Cells were cultured in Dulbecco’s Modified Eagle Medium (DMEM, Gibco, Thermo Fisher Scientific, Waltham, MA, USA) supplemented with 10% fetal bovine serum (FBS, Gibco, Thermo Fisher Scientific, Waltham, MA, USA) and maintained at 37 °C in a humidified incubator with 5% CO_2_. Cells were passaged 2–3 times per week using 0.25% trypsin-EDTA to maintain exponential growth and ensure consistent cell density for experiments. For all experimental procedures, cells were seeded at a density of 2 × 10^4^ or 1 × 10^5^ cells per well in culture plates appropriate for the downstream analyses: 96-well plates for cell proliferation and viability (WST-1 assay) and phase-contrast visualization and 6-well plates for the neutral Comet Assay. After seeding, cells were allowed to adhere for 24 h under standard culture conditions. Following attachment, cells were treated with four subcritical water extracts (SWE100/10, SWE125/10, SWE150/10, SWE170/10) and two conventional extracts (WM and TADEM) of *E. purpurea* at final concentrations of 100 μg/mL and 200 μg/mL. Extracts were freshly prepared in culture medium immediately before treatment, ensuring uniform exposure. Control cells received an equivalent volume of vehicle (medium without extracts). Cells were incubated for 24 h and 48 h post-treatment under standard conditions. At each time point, cells were processed according to the specific assay requirements. All experiments were performed in triplicate to ensure reproducibility, and cell viability was routinely monitored using trypan blue exclusion before treatment.

### 3.15. WST-1 Cell Proliferation Assay

The WST-1 assay (TaKaRa Bio INC., Kusatsu, Shiga, Japan) was used to evaluate the effects of the *E. purpurea* extracts on cell proliferation and viability after 24 h of supplementation, following a previously described protocol [83]. Cells were placed into 96-well plates at a density of 1 × 10^4^ cells per well and allowed to adhere for 24 h at 37 °C in a 5% CO_2_ atmosphere. The next day, the old culture medium was replaced with fresh medium, and the cells were incubated at increasing concentrations of the tested extracts for an additional 24 h. After this exposure period, the medium was replaced with fresh medium, and the WST-1 reagent was introduced directly to the cells at a 1:10 dilution, following the manufacturer’s guidelines. Following a 1 h incubation at 37 °C in the dark, the amount of formazan produced by the cells was measured by reading the absorbance at 450 nm using a standard microplate reader (INNO-S, LTEK, Gyeonggi-do, Republic of Korea). The optical density (OD) data from the cell proliferation assay were used to calculate cell viability as a percentage relative to the untreated control group.

### 3.16. Phase-Contrast Microscopy

Changes in cell morphology following 24 h and 48 h of treatment with the tested extracts were assessed using phase-contrast microscopy. Phase-contrast images were captured at 10× and 20× magnifications with an Axiovert 25 inverted microscope (Carl Zeiss Microscopy GmbH, Jena, Germany) fitted with a Leica K5C digital camera.

### 3.17. Comet Assay (Single-Cell Gel Electrophoresis, SCGE)

DNA damage at the single-cell level was evaluated using the neutral Comet Assay (SCGE) in HT-29 cells following exposure to the tested extracts. As a positive control, cells were treated with 250 mM hydrogen peroxide (H_2_O_2_) for 30 min to induce DNA damage, while untreated cells served as the negative control. After treatment, cells were harvested and resuspended, then mixed with 0.7% (*w*/*v*) low-melting-point agarose (Sigma-Aldrich) and immediately spread onto microscope slides pre-coated with a layer of normal-melting-point agarose to form microgels. The slides were allowed to solidify at 4 °C. Subsequently, the embedded cells were lysed in a cold lysis buffer containing 146 mM NaCl, 30 mM EDTA (pH 7.0), 10 mM Tris-HCl (pH 7.0), and 0.1% N-lauroylsarcosine for 20 min at 4 °C to remove cellular membranes and proteins while preserving nucleoids. Following lysis, slides were rinsed twice with 0.5× Tris-borate-EDTA (TBE) buffer to remove residual lysis components. Electrophoresis was then performed under neutral conditions at 0.46 V/cm for 10 min. After electrophoresis, DNA was stained with SYBR Green (Invitrogen, Carlsbad, CA, USA) and visualized using a fluorescence microscope. Comet images were analyzed quantitatively using CometScore software (Version 2.0, TriTek Corp., USA), and DNA damage was expressed as the Olive tail moment. All samples were processed under low-light conditions to prevent additional DNA damage.

### 3.18. Statistical Analysis

All extractions were performed in duplicate. The HPLC analyses were performed twice for every single sample (*n* = 4), whereas other analyses were run at least in triplicate for each sample (*n* = 6). Results are expressed as mean values ± standard deviations. Statistical testing involved one-way analysis of variance (ANOVA) followed by Tukey’s post hoc comparison test to determine significant differences between groups. A *p*-value of less than 0.05 (*p* < 0.05) was considered statistically significant. Microsoft Excel, 2013 (Microsoft Corporation, Redmond, WA, USA) was used in the analyses.

## 4. Conclusions

This study demonstrates that subcritical water extraction is an effective approach for producing *E. purpurea* root extracts standardized to cichoric and caftaric acid content. Extraction temperature was identified as the key factor affecting both hydroxycinnamic acid recovery and extract composition, with the most favorable conditions established at 110 °C for 10 min. The results reveal a clear distinction between the recovery of hydroxycinnamic acids and total polyphenols, demonstrating that optimization of SWE based solely on total polyphenol content is insufficient and may lead to misleading conclusions regarding the recovery of the target compounds. Increasing extraction temperature above 125 °C resulted in progressive thermal degradation of hydroxycinnamic acids despite the continued increase in total polyphenol content and extraction yield. Biological evaluation confirmed the favorable cytocompatibility of SWE-derived extracts and their comparable or superior performance relative to conventionally prepared extracts. The low content of 5-HMF in extracts obtained under mild extraction conditions (100–125 °C) provides an additional indication of their safety. Overall, SWE represents a promising green technology for the production of high-quality *E. purpurea* extracts for pharmaceutical, nutraceutical, food and cosmeceutical applications, achieving approximately 91% of the hydroxycinnamic acid recovery of conventional hydroethanolic extraction while requiring only 10 min extraction time and no organic solvents.

## Figures and Tables

**Table 1:** Proximate composition and antioxidant activity of three *E. purpurea* root batches.

	EP1	EP2	EP3
Moisture, %	4.3 ^b^ ± 0.1	6.5 ^a^ ± 0.2	6.3 ^a^ ± 0.1
Crude protein (N × 6.25), %	13.0 ^b^ ± 0.2	16.5 ^a^ ± 0.3	11.0 ^c^ ± 0.1
Total lipids, %	0.4 ^b^ ± 0.0	0.6 ^a^ ± 0.1	0.5 ^a^ ± 0.0
Total carbohydrates, %	52.1 ^a^ ± 3.0	43.0 ^b^ ± 1.1	49.5 ^a^ ± 4.2
Glucose (Glc)	0.1 ^a^ ± 0.0	0.1 ^a^ ± 0.0	0.1 ^a^ ± 0.1
Fructose (Fru)	5.1 ^b^ ± 0.1	5.8 ^a^ ± 0.2	2.9 ^c^ ± 0.1
Sucrose (Suc)	1.6 ^b^ ± 0.0	2.1 ^a^ ± 0.1	2.3 ^a^ ± 0.1
Total uronic acids	8.1 ^a^ ± 0.2	7.8 ^b^ ± 0.1	8.5 ^a^ ± 0.2
Cellulose	17.0 ^a^ ± 0.5	13.8 ^c^ ± 0.3	15.0 ^b^ ± 0.4
Total fructans	11.9 ^b^ ± 0.5	12.8 ^b^ ± 0.2	16.7 ^a^ ± 0.6
Starch	1.1 ^a^ ± 0.1	1.0 ^a^ ± 0.0	0.8 ^b^ ± 0.0
Ash, %	4.8 ^b^ ± 0.0	6.0 ^a^ ± 0.1	5.3 ^b^ ± 0.1
Lignin (ABSL), %	5.8 ^a^ ± 0.1	4.0 ^b^ ± 0.1	4.5 ^b^ ± 0.2
Phenolics, mg/100 g			
Total polyphenols	1376.4 ^b^ ± 18	1137.0 ^c^ ± 25	2516.5 ^a^ ± 15
Hydroxycinnamic acids, %			
Caftaric acid	0.09 ^b^ ± 0.01	0.10 ^b^ ± 0.02	0.25 ^a^ ± 0.01
Cichoric acid	0.82 ^b^ ± 0.05	0.59 ^c^ ± 0.06	1.92 ^a^ ± 0.05
Sum	0.91	0.69	2.17
Antioxidant activity			
ORAC, µmol TE/g DW	431.6 ^c^ ± 5.0	457.7 ^b^ ± 4.0	672.5 ^a^ ± 10.0
HORAC, µmol GAE/g DW	90.4 ^b^ ± 2.5	94.4 ^b^ ± 1.5	139.3 ^a^ ± 3.0

Results are presented as mean values ± SD. There are no significant differences among values marked with the same letters (a, b, c) within individual rows (*p* < 0.05). EP1—*E. purpurea* 1, EP2—*E. purpurea* 2 and EP3—*E. purpurea* 3. ORAC—oxygen radical absorbance capacity, HORAC—hydroxyl radical averting capacity.

**Table 2:** Content of individual phenolics and antioxidant activity of freeze-dried extracts, obtained by different extraction methods.

Extract	Gallic Acid, mg/100 g DW	Chlorogenic Acid, mg/100 g DW	Caffeic Acid, mg/100 g DW	Rutin, mg/100 g DW	ORAC, µmol TE/g DW
SWE100/10	44.1 ^h^ ± 3.6	132.3 ^a^ ± 10.4	72.7 ^g^ ± 6.4	28.6 ^f^ ± 2.1	3340 ^c^ ± 158
SWE100/20	48.0 ^g^ ± 4.1	139.9 ^a^ ± 11.6	114.5 ^f^ ± 10.1	51.1 ^d^ ± 4.8	3369 ^c^ ± 162
SWE100/30	47.1 ^g^ ± 3.9	122.7 ^a^ ± 11.8	104.5 ^f^ ± 9.8	40.8 ^e^ ± 3.4	3252 ^c^ ± 143
SWE125/10	53.8 ^f^ ± 4.6	138.3 ^a^ ± 12.6	255.7 ^e^ ± 20.3	92.7 ^c^ ± 8.6	3238 ^c^ ± 138
SWE125/20	67.3 ^e^ ± 5.7	133.4 ^a^ ± 11.2	387.1 ^d^ ± 30.6	147.3 ^b^ ± 12.1	3485 ^c^ ± 161
SWE125/30	72.5 ^d^ ± 6.1	133.1 ^a^ ± 11.4	427.7 ^c^ ± 38.2	169.7 ^b^ ± 13.3	3259 ^c^ ± 136
SWE150/10	122.8 ^c^ ± 10.6	128.8 ^a^ ± 10.2	945.7 ^a^ ± 89.1	440.9 ^a^ ± 42.3	3487 ^b^ ± 156
SWE150/20	119.9 ^c^ ± 10.1	36.7 ^b^ ± 2.7	695.7 ^b^ ± 54.2	458.5 ^a^ ± 42.7	4036 ^a^ ± 248
SWE150/30	118.9 ^c^ ± 9.8	21.9 ^d^ ± 1.1	380.8 ^d^ ± 30.2	454.3 ^a^ ± 41.8	4238 ^a^ ± 226
SWE170/10	121.6 ^c^ ± 11.3	10.3 ^f^ ± 0.9	474.4 ^c^ ± 39.2	461.7 ^a^ ± 43.0	3851 ^a^ ± 201
SWE170/20	175.5 ^a^ ± 14.1	23.7 ^d^ ± 2.1	18.7 ^j^ ± 1.2	143.9 ^b^ ± 13.8	4238 ^a^ ± 234
SWE170/30	157.4 ^b^ ± 12.3	31.2 ^c^ ± 2.8	8.8 ^k^ ± 0.2	85.4 ^c^ ± 7.8	3715 ^b^ ± 198
TADEM	40.5 ^h^ ± 3.4	27.3 ^d^ ± 1.2	46.4 ^h^ ± 3.4	23.2 ^g^ ± 1.9	3732 ^b^ ± 167
WM	41.3 ^h^ ± 3.0	19.9 ^e^ ± 0.9	24.0 ^i^ ± 2.1	9.3 ^h^ ± 0.3	2483 ^d^ ± 120

Results are presented as mean values ± SD. There are no significant differences among values marked with the same letters (a, b, c, etc.) within individual columns (*p* < 0.05).

**Table 3:** Yield and proximate composition analysis of residue after subcritical water extraction (*w*/*w*%).

Constituents	SWE100/10	SWE100/20	SWE150/10	SWE150/20
Yield of cell wall material, %	77.1 ^b^ ± 1.0	82.7 ^a^ ± 2.0	76.8 ^b^ ± 0.5	79.8 ^a^ ± 1.5
Yield of residue, %	71.7 ^a^ ± 1.5	65.9 ^b^ ± 1.2	55.3 ^c^ ± 0.5	51.0 ^d^ ± 1.0
Crude protein (N × 6.25)	12.3 ^b^ ± 0.1	13.5 ^a^ ± 0.2	13.4 ^a^ ± 0.2	11.9 ^b^ ± 0.0
Recovery, %	55.0	55.6	46.2	37.7
Total uronic acids	11.8 ^a^ ± 0.2	11.9 ^a^ ± 0.1	8.1 ^b^ ± 0.1	6.1 ^c^ ± 0.0
Recovery, %	91.2	84.7	48.6	33.7
Cellulose	20.9 ^b^ ± 0.3	21.2 ^b^ ± 0.2	27.7 ^a^ ± 0.1	28.1 ^a^ ± 0.2
Recovery, %	105.2	97.9	107.7	100.7
Total fructans, %	6.2 ^a^ ± 0.2	3.9 ^b^ ± 0.1	3.2 ^c^ ± 0.2	2.2 ^d^ ± 0.1
Recovery, %	36.6	21.3	14.6	9.4
Lignin (Klason)	17.0 ^b^ ± 0.0	16.9 ^b^ ± 0.0	19.7 ^a^ ± 1.0	20.8 ^a^ ± 0.5
Recovery, %	101.1	92.6	90.3	88.2

Results are presented as mean values ± SD. There are no significant differences among values marked with the same letters (a, b, c, d) within individual rows (*p* < 0.05).

**Table 4:** Total carbohydrate, uronic acids, 5-(hydroxymethyl)furfural (5-HMF) content, sugar and organic acids composition of dried purple coneflower root subcritical water extracts.

Constituents	SWE 100/10	SWE 100/20	SWE 100/30	SWE 125/10	SWE 125/20	SWE 125/30	SWE 150/10	SWE 150/20	SWE 150/30	SWE 170/10	SWE 170/20	SWE 170/30
Total carbohydrates, %	42.4 ^e^ ± 0.6	50.7 ^b^ ± 1.0	48.0 ^c^ ± 0.5	43.2 ^e^ ± 0.4	50.3 ^b^ ± 0.4	57.6 ^a^ ± 1.2	40.5 ^f^ ± 0.5	50.1 ^b^ ± 0.7	43.2 ^d^ ± 0.4	42.2 ^e^ ± 0.5	42.3 ^e^ ± 0.4	42.5 ^e^ ± 0.5
Total uronic acids, %	2.8 ^f^ ± 0.1	4.3 ^c^ ± 0.3	3.8 ^d^ ± 0.2	4.6 ^b^ ± 0.4	6.2 ^a^ ± 0.5	5.3 ^b^ ± 0.4	4.6 ^c^ ± 0.2	4.3 ^c^ ± 0.3	3.3 ^e^ ± 0.1	3.0 ^e^ ± 0.1	2.6 ^f^ ± 0.0	2.5 ^f^ ± 0.0
Recovery, % (100—%)	8.2 (91.8)	14.1 (85.9)	12.9 (87.1)	15.1 (84.9)	31.8 (68.2)	23.5 (76.5)	20.0 (80.0)	20.2 (79.8)	16.3 (83.7)	9.3 (90.7)	11.9 (88.1)	11.9 (88.1)
Glucose (Glc)	0.8 ^e^ ± 0.0	1.4 ^d^ ± 0.2	1.1 ^d^ ± 0.0	1.4 ^d^ ± 0.1	1.4 ^d^ ± 0.1	1.6 ^d^ ± 0.1	3.0 ^c^ ± 0.3	5.3 ^a^ ± 0.4	4.5 ^b^ ± 0.3	4.2 ^b^ ± 0.3	3.8 ^bc^ ± 0.2	3.5 ^c^ ± 0.2
Fructose (Fru)	7.1 ^g^ ± 0.2	7.3 ^fg^ ± 0.6	6.8 ^g^ ± 0.3	8.3 ^f^ ± 0.3	10.5 ^e^ ± 0.2	13.1 ^d^ ± 0.4	26.7 ^b^ ± 0.7	32.1 ^a^ ± 1.0	27.0 ^b^ ± 0.7	19.2 ^c^ ± 0.6	12.6 ^d^ ± 0.5	9.8 ^e^ ± 0.2
Sucrose (Suc)	5.9 ^a^ ± 0.4	5.6 ^b^ ± 0.4	5.0 ^b^ ± 0.2	6.5 ^a^ ± 0.3	5.4 ^b^ ± 0.3	5.2 ^b^ ± 0.2	5.1 ^b^ ± 0.1	2.0 ^c^ ± 0.1	0.3 ^d^ ± 0.1	0.3 ^d^ ± 0.0	0.1 ^e^ ± 0.0	0.1 ^e^ ± 0.1
Total	13.7	14.3	12.9	16.3	17.4	19.9	34.8	39.4	31.7	23.7	16.5	13.3
5-HMF (mg/100 g)	n.f.	n.f.	n.f.	6.1 ^h^ ± 1.1	25.0 ^g^ ± 2.1	30.2 ^f^ ±1 .3	101.1 ^e^ ± 5.2	125.2 ^d^ ± 3.2	139.0 ^c^ ± 4.3	168.1 ^b^ ± 4.5	201.0 ^a^ ± 9.1	198.2 ^a^ ± 6.1
Organic acids, %												
Malic acid	1.5 ^e^ ± 0.2	1.6 ^e^ ± 0.1	1.5 ^e^ ± 0.3	2.7 ^d^ ± 0.2	3.6 ^d^ ± 0.5	4.6 ^c^ ± 0.6	7.1 ^b^ ± 0.5	7.0 ^b^ ± 0.4	7.7 ^b^ ± 0.2	5.6 ^c^ ± 0.2	7.0 ^b^ ± 0.5	9.0 ^a^ ± 0.7
Acetic acid	1.2 c ± 0.0	1.0 c ± 0.1	1.0 c ± 0.0	1.0 c ± 0.2	1.0 c ± 0.1	1.0 c ± 0.2	1.6 b ± 0.1	2.0 a ± 0.3	2.2 a ± 0.2	2.0 a ± 0.3	2.1 a ± 0.1	2.1 a ± 0.0
Oxalic acid	0.9 ^a^ ± 0.1	0.9 ^a^ ± 0.0	0.8 ^a^ ± 0.2	0.7 ^ab^ ± 0.1	0.5 ^b^ ± 0.1	0.4 ^b^ ± 0.0	0.2 ^c^ ± 0.0	0.1 ^c^ ± 0.0	0.1 ^c^ ± 0.0	0.1 ^c^ ± 0.1	0.1 ^c^ ± 0.1	0.1 ^c^ ± 0.0
Citric acid	0.3 ^b^ ± 0.1	0.2 ^b^ ± 0.0	0.2 ^b^ ± 0.2	0.3 ^b^ ± 0.2	0.4 ^b^ ± 0.1	0.4 ^b^ ± 0.0	1.1 ^a^ ± 0.2	1.4 ^a^ ± 0.2	1.4 ^a^ ± 0.4	1.4 ^a^ ± 0.3	1.6 ^a^ ± 0.3	1.6 ^a^ ± 0.3
Total	3.9	3.7	3.6	4.8	5.5	6.4	9.9	10.6	11.4	9.2	10.8	12.8

Results are presented as mean values ± SD. There are no significant differences among values marked with the same letters (a, b, c, d, etc.) within individual rows (*p* < 0.05). n.f.—not found.

**Table 5:** Extraction conditions applied for subcritical water and conventional extraction of *E. purpurea* roots.

Extraction	Solvent	Temperature	Time	L/S Ratio
SWE100/10	water	100 °C	10 min	1:20
SWE100/20	water	100 °C	20 min	1:20
SWE100/30	water	100 °C	30 min	1:20
SWE110/10	water	110 °C	10 min	1:20
SWE125/10	water	125 °C	10 min	1:20
SWE125/20	water	125 °C	20 min	1:20
SWE125/30	water	125 °C	30 min	1:20
SWE150/10	water	150 °C	10 min	1:20
SWE150/20	water	150 °C	20 min	1:20
SWE150/30	water	150 °C	30 min	1:20
SWE170/10	water	170 °C	10 min	1:20
SWE170/20	water	170 °C	20 min	1:20
SWE170/30	water	170 °C	30 min	1:20
Water maceration (WM)	water	100 °C	45 min	1:20
Temperature-assisted dynamic ethanol maceration (TADEM)	70% ethanol	60 °C	60 min	1:20

## Data Availability

The original contributions presented in this study are included in the article. Further inquiries can be directed to the corresponding author.

## Abbreviations

The following abbreviations are used in this manuscript:

HORAC	Hydroxyl radical averting capacity
ORAC	Oxygen radical absorbance capacity
SWE	Subcritical water extraction
TADEM	Temperature-Assisted Dynamic Ethanol Maceration
WM	Static Water Maceration
GOPOD	glucose oxidase/peroxidase/4-aminoantipyrine
ABL	acetyl bromide lignin
5-HMF	5-(hydroxymethyl)furfural
